# Food Security and Cardiovascular Disease Risk Among Adults in the United States: Findings From the National Health and Nutrition Examination Survey, 2003–2008

**DOI:** 10.5888/pcd10.130244

**Published:** 2013-12-05

**Authors:** Earl S. Ford

## Abstract

**Introduction:**

Little is known about the relationship between food security status and predicted 10-year cardiovascular disease risk. The objective of this study was to examine the associations between food security status and cardiovascular disease risk factors and predicted 10-year risk in a national sample of US adults.

**Methods:**

A cross-sectional analysis using data from 10,455 adults aged 20 years or older from the National Health and Nutrition Examination Survey 2003–2008 was conducted. Four levels of food security status were defined by using 10 questions.

**Results:**

Among all participants, 83.9% had full food security, 6.7% had marginal food security, 5.8% had low food security, and 3.6% had very low food security. After adjustment, mean hemoglobin A1c was 0.15% greater and mean concentration of C-reactive protein was 0.8 mg/L greater among participants with very low food security than among those with full food security. The adjusted mean concentration of cotinine among participants with very low food security was almost double that of participants with full food security (112.8 vs 62.0 ng/mL, *P* < .001). No significant associations between food security status and systolic blood pressure or concentrations of total cholesterol, high-density lipoprotein cholesterol, or non-high-density lipoprotein cholesterol were observed. Participants aged 30 to 59 years with very low food security were more likely to have a predicted 10-year cardiovascular disease risk greater than 20% than fully food secure participants (adjusted prevalence ratio, 2.38; 95% CI, 1.31–4.31).

**Conclusion:**

Adults aged 30 to 59 years with very low food security showed evidence of increased predicted 10-year cardiovascular disease risk.

## Introduction

Despite the status of the United States as a wealthy country, questions about food security during the 1980s led to a concerted effort to more clearly define the state of US food security (1,2). The US Department of Agriculture defines food security as “access by all members at all times to enough food for an active, healthy life (3).” Since 1995, food security has generally been measured with a standard set of questions about a person’s ability to afford food and about possible consequences of inadequate funds, such as reduced food intake, hunger, and weight loss. The most recent data from the Current Population Survey in 2011 showed that 85.1% of households were fully food secure and 14.9% were food insecure (4).

Little is known about the associations between food security and predicted cardiovascular disease risk, but some research has addressed associations between food security and several cardiovascular disease risk factors. Although studies have linked food insecurity to obesity in adults, especially among women, the evidence among adults as well as children and adolescents is unsettled (5,6). The prevalence of diabetes has been shown to be higher among food-insecure adults than food-secure adults in 2 national surveys (7,8). Furthermore, food-insecure adults with diabetes had poorer glycemic control than food-secure adults with diabetes (9,10). A cross-sectional analysis of national data showed that the prevalence of metabolic syndrome was higher among participants with marginal and very low food security than among fully food secure participants (11). Diets of food-insecure adults may be of low quality (12), which in turn could affect cardiometabolic risk factors for cardiovascular disease. Finally, several studies have noted that the prevalence of smoking was higher among food-insecure adults than among food-secure adults (13–15). In contrast, other studies have not found differences in diastolic blood pressure, hyperlipidemia, and concentrations of total cholesterol, blood glucose, and hemoglobin A1c (HbA1c) by food security status (8,16). Consequently, these considerations suggest that food insecurity could be associated with increased cardiovascular disease risk. Therefore, the objective of this study was to examine the cross-sectional associations between cardiovascular disease risk factors, cardiovascular disease risk, and food security among US adults.

## Methods

Data from the National Health and Nutrition Examination Survey (NHANES) 2-year cycles conducted from 2003 through 2008 were used in this analysis (17). During each 2-year cycle, NHANES employed a multistage, stratified sampling design to select a national sample that yielded results representative of the civilian, noninstitutionalized US population. Trained interviewers administered study questionnaires to participants in their homes and extended an invitation to participants to have an examination at a mobile center where they were asked to undergo various examinations, provide a blood sample, and complete additional questionnaires. The response rates were 79% for NHANES 2003–2004, 80% for NHANES 2005–2006, and 78% for NHANES 2007–2008 for the interviewed samples and 76% for NHANES 2003–2004, 77% for NHANES 2005–2006, and 75% for NHANES 2007–2008 for the examined samples. The National Center for Health Statistics Research Ethics Review Board granted approval for the surveys, and participants were asked to sign an informed consent form.

The food security questions used in this study represented the culmination of efforts to develop a set of questions to assess food insecurity in the United States. A description of the historical events that led to the development of the food security questionnaire, the early application of these questions in other surveys such as the Current Population Survey, and the psychometric properties of the questions are recounted elsewhere (2). Adult food security was determined from a series of 10 questions (Appendix) (18,19). Four levels were established: full food security (0 points), marginal food security (1–2 points), low food security (3–5 points), and very low food security (6–10 points).

Tests to assess cardiovascular disease risk factors included HbA1c, systolic blood pressure, total cholesterol, high-density lipoprotein (HDL) cholesterol, non-HDL cholesterol, smoking status, cotinine, C-reactive protein, body mass index (BMI), and urinary albumin–creatinine ratio. Measurements of these variables have been described elsewhere (17).

The cardiovascular disease risk factors were dichotomized as follows: diabetes (HbA1c ≥6.5% or the use of insulin or oral hypoglycemic medications), hypertension (systolic blood pressure ≥140 mm Hg or diastolic blood pressure) ≥90 mm Hg or the self-reported current use of antihypertensive medications), high total cholesterol (≥200 mg/dl [5.17 mmol/L]), low HDL cholesterol (<40 mg/dL [1.03 mmol/L] in men and <50 mg/dL [1.29 mmol/L] in women), high non-HDL cholesterol (≥130 mg/dL [<3.36 mmol/L]), high C-reactive protein (>3 mg/L), and high urinary albumin–creatinine ratio (≥30 mg/g). Participants who responded affirmatively to the question “Have you ever been told by a doctor or health professional you have diabetes or sugar diabetes?” were asked about the use of insulin or oral hypoglycemic medications.

Predicted 10-year cardiovascular disease risk was determined from a multivariable risk algorithm derived from Framingham data (20). This algorithm includes age, concentrations of total cholesterol and HDL cholesterol, systolic blood pressure, hypertension treatment status, smoking, and diabetes. To calculate predicted 10-year cardiovascular disease risk, participants who reported using insulin or oral hypoglycemic medications or had a concentration of fasting plasma glucose at or greater than 126 mg/dL were considered to have diabetes. Participants attended either a morning examination during which fasting blood specimens were collected or an afternoon or evening examination. Because the risk equation uses diabetes defined on the basis of fasting plasma concentrations of blood glucose or the use of insulin or oral hypoglycemic medications, the calculation of predicted 10-year cardiovascular disease risk was limited to the subsample of participants who attended the morning examination.

Additional variables included in the analyses were age, sex, race/ethnicity (white, African American, Mexican American, and other race or ethnicity), education (<high school, high school diploma or general equivalency diploma, and >high school), alcohol use, and health insurance status (yes/no). Alcohol use was operationalized as never had 12 drinks during lifetime or any given year, had 12 or more drinks during lifetime but not in any given year, had 12 or more drinks in any given year but did not use alcohol during past year, moderate use (≤1 drink per day in women and ≤2 drinks per day in men), and excessive use (>1 drink per day in women and >2 drinks per day in men).

The analyses were limited to participants aged 20 years or older and free of self-reported cardiovascular disease (ie, heart failure, coronary heart disease, angina, myocardial infarction, and stroke). The direct method was used to adjust for age by using the projected year 2000 US population. The significance of associations between food security status and cardiovascular disease risk factors, which were examined with linear regression analyses for continuous variables and with log-linear regression for categorical variables, was tested with the adjusted Wald F test and Wald χ^2^ test, respectively. Model 1 included age, sex, race/ethnicity, educational status, health insurance coverage, and alcohol use. Model 2 added HbA1c, systolic blood pressure, total cholesterol, HDL cholesterol, non-HDL cholesterol, BMI, cotinine, C-reactive protein, and urinary albumin–creatinine ratio. Because the literature suggests that the associations between food security status and obesity differ by sex, a stratified analysis examining the association between food security status and BMI and obesity was performed. Unweighted numbers for sample sizes are shown. All estimates were calculated using the sampling weights. SUDAAN (Software for the Statistical Analysis of Correlated Data) (Research Triangle Institute, Research Triangle Park, North Carolina) was used for the analyses to account for the complex sampling design.

## Results

Of the 15,222 participants aged 20 years or older who attended the mobile examination center, food security status was established for 14,947 participants. After excluding pregnant women, participants with a history of cardiovascular disease, and participants with missing values for the other study variables, 10,455 participants were included in the analytic sample. The sample included 5,253 men, 5,202 women, 5,262 whites, 2,068 African Americans, 2,059 Mexican Americans, and 1,066 participants of another race or ethnicity. The median age was 43.8 years; 16.8% had not graduated from high school, 25.2% had graduated from high school or completed its equivalent, and 58.0% had received education beyond high school.

Among all participants, 83.9% (standard error [SE], 0.6) were fully food secure, 6.7% (SE, 0.4) had marginal food security, 5.8% (SE, 0.3) had low food security, and 3.6% (SE, 0.3) had very low food security. The unadjusted distribution of food security status among participants who were excluded from the study (80.3%, SE, 1.3; 9.3%, SE, 0.9; 6.8%, SE, 0.7; 3.6%, SE, 0.5, respectively) differed significantly from that of participants who were included (*P* = .03). The distribution of food security status did not differ by sex (*P* = .23) but did differ by race/ethnicity (*P* <.001). The median age was 45.1 years for participants with full food security, 37.0 years for participants with marginal food security, 37.3 years for participants with low food security, and 39.1 years for participants with very low food security.

In the model adjusted for age, sex, race/ethnicity, educational status, health insurance coverage, and alcohol use, significant associations were observed for BMI and concentrations of HbA1C, HDL cholesterol, cotinine, and C-reactive protein. Of the 4 groups, participants with very low food security generally had the poorest levels. With additional adjustment, food security status was still significantly associated with concentrations of HbA1c, cotinine, and C-reactive protein (Table 1). In models with maximal adjustment, mean concentration of HbA1c was 0.15% higher and mean concentration of C-reactive protein was 0.8 mg/L greater among participants with very low food security than among those with full food security. The mean concentration of cotinine among participants with very low food security was almost double that of those with full food security.

**Table 1:** Least-Square Adjusted Means of Risk Factors for Cardiovascular Disease Among Participants (N = 10,455) Aged 20 Years or Older, by Food Security Status, National Health and Nutrition Examination Surveys, 2003–2008

Risk Factor^a^	Food Security Status				*P* Value^b^
	Full (n = 8,145)	Marginal (n = 976)	Low (n = 857)	Very Low (n = 477)	
Model 1^c^					
HbA1c	5.45 (0.01)	5.50 (0.04)	5.54 (0.03)	5.64 (0.08)	.006
Systolic blood pressure, mm Hg	121.3 (0.3)	121.5 (0.7)	120.5 (0.8)	122.9 (0.8)	.18
Total cholesterol, mg/dL	199.5 (0.6)	201.5 (1.6)	200.4 (1.7)	202.1 (2.6)	.61
High-density lipoprotein cholesterol, mg/dL	53.8 (0.2)	51.9 (0.6)	52.7 (0.8)	52.4 (0.9)	<.001
Non-high-density lipoprotein cholesterol, mg/dL	145.7 (0.6)	149.7 (1.5)	147.7 (2.0)	149.7 (2.6)	.06
Body mass index, kg/m^2^	28.2 (0.1)	29.0 (0.3)	28.6 (0.3)	29.0 (0.6)	.01
Cotinine, ng/mL	61.9 (2.1)	84.1 (6.0)	89.7 (7.7)	113.4 (9.4)	<.001
C-reactive protein, mg/L	3.9 (0.1)	4.0 (0.2)	3.8 (0.2)	5.1 (0.5)	.02
Urinary albumin–creatinine ratio, mg/g	22.3 (1.9)	25.9 (4.4)	30.1 (7.8)	33.8 (10.2)	.57
**Model 2^d^**					
HbA1c	5.45 (0.01)	5.47 (0.03)	5.53 (0.03)	5.61 (0.08)	.04
Systolic blood pressure, mm Hg	121.3 (0.3)	121.2 (0.7)	120.4 (0.7)	122.6 (0.8)	.32
Total cholesterol, mg/dL	199.6 (0.6)	201.4 (1.6)	200.2 (1.7)	200.8 (2.6)	.73
High-density lipoprotein cholesterol, mg/dL	53.7 (0.2)	52.5 (0.5)	53.2 (0.8)	53.5 (0.8)	.06
Non-high-density lipoprotein cholesterol, mg/dL	146.0 (0.6)	148.5 (1.5)	146.8 (2.0)	147.2 (2.6)	.43
Body mass index, kg/m^2^	28.2 (0.1)	28.7 (0.2)	28.6 (0.3)	28.6 (0.5)	.10
Cotinine, ng/mL	62.0 (2.2)	83.8 (6.1)	89.3 (7.6)	112.8 (9.0)	<.001
C-reactive protein, mg/L	3.9 (0.1)	3.8 (0.2)	3.6 (0.2)	4.8 (0.4)	.04
Urinary albumin-creatinine ratio, mg/g	22.7 (2.0)	25.0 (4.4)	29.3 (7.3)	27.3 (10.3)	.81

Abbreviation: HbA1c, hemoglobin A1c.

a Values are mean (standard error), unless otherwise indicated.

b Calculated by using adjusted Wald F test.

c Model 1 is adjusted for age, sex, race/ethnicity, educational status, health insurance coverage, and alcohol use.

d Model 2 is adjusted for variables in Model 1 plus other cardiovascular disease risk factors shown in this table.

Numerous associations between the age-adjusted prevalence of dichotomized risk factors and food security status were present (Table 2). After maximal adjustment, prevalence ratios indicated significant associations for smoking status and hypercotinemia and for urinary albumin–creatinine ratio less than 30 mg/g (Table 2). Simply adding BMI to Model 1 attenuated the significant association between food security status and diabetes.

**Table 2:** Age-Adjusted Prevalence (% [SE]) of Abnormal Risk Factors for Cardiovascular Disease and Adjusted Prevalence Ratios (95% CI) for Abnormal Risk Factors for Cardiovascular Disease Among Participants (N = 10,455) Aged 20 years or Older, National Health and Nutrition Examination Surveys, 2003–2008

Risk Factor	Food Security Status				*P* Value^a^
	Full (n = 8,145)	Marginal (n = 976)	Low (n = 857)	Very low (n = 477)	
Prevalence					
HbA1c ≥6.5% or use of insulin or hypoglycemic oral medications	7.1 (0.4)	11.5 (1.5)	10.6 (1.3)	14.6 (2.4)	<.001
Hypertension	28.4 (0.6)	31.8 (2.2)	27.9 (2.1)	36.8 (3.0)	.001
Current smoker	21.5 (0.8)	32.7 (1.8)	32.9 (2.0)	45.9 (3.2)	<.001
Cotinine >10 ng/mL	25.7 (1.0)	35.0 (2.1)	35.1 (1.9)	50.1 (3.2)	<.001
Total cholesterol ≥200 mg/dL or use of cholesterol-lowering medications	53.9 (0.7)	55.1 (2.0)	52.0 (1.9)	54.7 (2.5)	.96
Low high-density lipoprotein cholesterol	27.2 (0.8)	34.2 (1.8)	31.9 (2.0)	34.8 (2.3)	<.001
Non-high-density lipoprotein cholesterol ≥130 mg/dL	62.3 (0.7)	65.9 (2.1)	60.9 (2.3)	62.4 (2.7)	.65
Body mass index ≥30 kg/m^2^	31.2 (0.8)	38.2 (1.7)	36.1 (2.0)	36.3 (3.0)	.001
C-reactive protein >3 mg/L	31.5 (0.7)	40.9 (1.7)	34.9 (2.0)	42.4 (3.5)	<.001
Urinary albumin–creatinine ratio ≥30 mg/g	8.0 (0.3)	10.9 (1.4)	14.1 (1.5)	11.7 (2.0)	<.001
**Adjusted Prevalence Ratio**					
**Model 1^b^**					
HbA1c ≥6.5% or use of insulin or hypoglycemic oral medications	1 [Reference]	1.18 (0.90–1.54)	1.09 (0.83–1.43)	1.84 (1.26–2.69)	.01
Hypertension		1.08 (0.94–1.25)	0.92 (0.77–1.11)	1.38 (1.11–1.71)	.004
Current smoker		1.36 (1.20–1.54)	1.38 (1.23–1.54)	1.66 (1.46–1.88)	<.001
Cotinine >10 ng/mL		1.28 (1.13–1.45)	1.27 (1.15–1.40)	1.54 (1.37–1.72)	<.001
Total cholesterol ≥200 mg/dL or use of cholesterol-lowering medications		1.03 (0.93–1.13)	1.02 (0.93–1.11)	1.05 (0.94–1.17)	.76
Low high-density lipoprotein cholesterol		1.04 (0.97–1.11)	1.00 (0.93–1.07)	1.00 (0.90–1.10)	.002
Non-high-density lipoprotein cholesterol ≥130 mg/dL		1.18 (1.06–1.32)	1.10 (0.97–1.26)	1.20 (1.02–1.41)	.73
Body mass index ≥30 kg/m^2^		1.15 (1.03–1.28)	1.10 (0.98–1.23)	1.14 (0.96–1.35)	.03
C-reactive protein >3 mg/L		1.17 (1.06–1.28)	1.07 (0.95–1.21)	1.25 (1.03–1.51)	.002
Urinary albumin-creatinine ratio ≥30 mg/g		1.27 (0.96–1.67)	1.41 (1.13–1.75)	1.49 (1.01–2.20)	.01
**Model 2^c^**					
HbA1c ≥6.5% or use of insulin or hypoglycemic oral medications	1 [Reference]	1.11 (0.85–1.44)	1.02 (0.78–1.34)	1.37 (0.89–2.10)	.48
Hypertension		1.04 (0.90–1.19)	0.88 (0.75–1.04)	1.21 (0.98–1.50)	.08
Current smoker		1.33 (1.17–1.50)	1.36 (1.22–1.51)	1.61 (1.43–1.82)	<.001
Cotinine >10 ng/mL		1.25 (1.10–1.42)	1.25 (1.14–1.37)	1.50 (1.34–1.68)	<.001
Total cholesterol ≥200 mg/dL or use of cholesterol-lowering medications		1.01 (0.92–1.12)	1.00 (0.92–1.09)	1.01 (0.91–1.13)	.99
Low high-density lipoprotein cholesterol		1.12 (1.01–1.24)	1.05 (0.92–1.20)	0.99 (0.80–1.23)	.18
Non-high-density lipoprotein cholesterol ≥130 mg/dL		1.02 (0.96–1.08)	0.98 (0.91–1.06)	0.96 (0.87–1.05)	.73
Body mass index ≥30 kg/m^2^		1.11 (1.00–1.22)	1.08 (0.96–1.21)	1.04 (0.89–1.22)	.17
C-reactive protein >3 mg/L		1.06 (0.9–1.17)	0.98 (0.87–1.10)	0.98 (0.82–1.18)	.25
Urinary albumin–creatinine ratio ≥30 mg/g		1.26 (0.97–1.63)	1.32 (1.06–1.65)	1.24 (0.86–1.79)	.04

Abbreviations: SE, standard error; CI, confidence interval; HbA1c, hemoglobin A1c.

a Calculated by using Wald χ^2^ test.

b Adjusted for age, sex, race/ethnicity, educational status, health insurance coverage, and alcohol use.

c Adjusted for variables in Model 1 plus HbA1c, systolic blood pressure, total cholesterol, high-density lipoprotein cholesterol, body mass index, cotinine, C-reactive protein, and urinary albumin–creatinine ratio.

 Among women, food security status was significantly associated with BMI; no significant associations were found among men (Table 3). Depending on the model and category of food insecurity, food-insecure women had a higher BMI, ranging from 1.0 to 1.7 kg/m^2^, than did food-secure women. Furthermore, obesity was from 21% to 35% higher among food-insecure women than among food-secure women.

**Table 3:** Least-Square Adjusted Means (SE) for Body Mass Index and Adjusted Prevalence Ratios (95% CI) Among Participants (N = 10,455) Aged 20 Years or Older, by Sex and Food-Security Status, National Health and Nutrition Examination Surveys, 2003–2008

Variable	Food Security Status				*P* Value^a^
	Full	Marginal	Low	Very low	
**Body Mass Index, kg/m^2^**					
**Men (n = 5,253)**					
Model 1^b^	28.4 (0.1)	28.4 (0.4)	27.9 (0.4)	28.2 (0.6)	.59
Model 2^c^	28.4 (0.1)	28.2 (0.3)	28.0 (0.3)	28.1 (0.6)	.62
**Women (n = 5,202)**					
Model 1^b^	28.0 (0.2)	29.4 (0.3)	29.2 (0.4)	29.7 (0.7)	<.001
Model 2^c^	28.0 (0.1)	29.2 (0.3)	29.1 (0.4)	29.1 (0.5)	<.001
**Obesity^d^**					
**Men (n = 5,253)**					
Model 1^b^	1 [Reference]	1.01 (0.84–1.22)	0.86 (0.71–1.04)	0.89 (0.71–1.13)	.39
Model 2^c^	1 [Reference]	0.97 (0.82–1.15)	0.88 (0.74–1.05)	0.88 (0.71–1.08)	.37
**Women (n = 5,202)**					
Model 1^b^	1 [Reference]	1.26 (1.10–1.43)	1.29 (1.13–1.47)	1.33 (1.07–1.66)	<.001
Model 2^c^	1 [Reference]	1.23 (1.08–1.40)	1.26 (1.09–1.47)	1.17 (0.97–1.42)	<.001

Abbreviations: SE, standard error; CI, confidence interval; HbA1c, hemoglobin A1c.

a Calculated by using adjusted Wald F test or Wald χ^2^ test.

b Adjusted for age, race/ethnicity, educational status, health insurance coverage, and alcohol use.

c Adjusted for variables in Model 1 plus HbA1c, systolic blood pressure, total cholesterol, high-density lipoprotein cholesterol, cotinine, C-reactive protein, and urinary albumin–creatinine ratio.

d Defined as body mass index ≥30 kg/m^2^.

In the fasting subsample of 3,446 participants aged 30 to 74 years, the unadjusted distribution of predicted 10-year risk for cardiovascular disease varied significantly by food security status (full food security 10.3% [SE, 0.7]; marginal food security 8.3% [SE, 1.6]; low food security 8.3% [SE, 1.3]; very low food security 16.4% [SE, 3.1]; *P* = .04). However, no significant association was observed between food security status and the predicted 10-year cardiovascular disease risk greater than 20% after adjustment for race/ethnicity, educational status, health insurance status, alcohol use, BMI, and concentration of C-reactive protein (*P* = .14). Stratified analyses by age group showed that food security status was significantly associated with the predicted 10-year risk greater than 20% among adults aged 30 to 59 years (*P* = .03) but not among those aged 60 to 74 years (*P* = .43) after adjustment for race/ethnicity, educational status, health insurance status, alcohol use, BMI, and concentration of C-reactive protein (Figure). The adjusted prevalence ratio among participants aged 30 to 59 years who had very low food security status was 2.38 (95% confidence interval [CI], 1.31–4.31).

**Figure:**
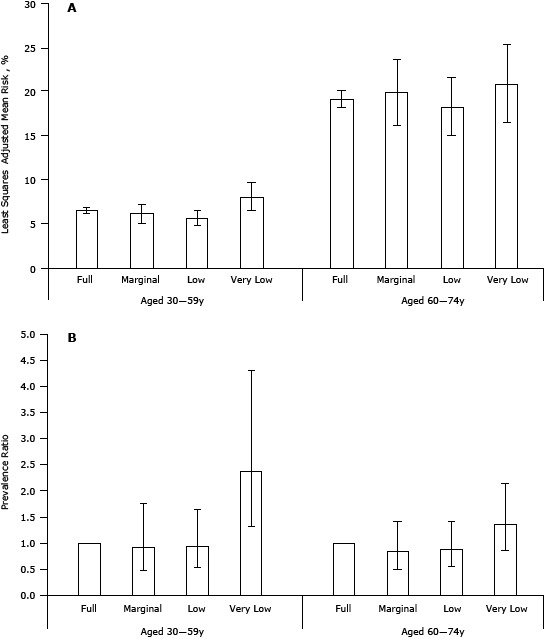
Least-square adjusted mean (95% confidence interval) predicted 10-year cardiovascular disease risk (Panel A) and adjusted prevalence ratios (95% confidence interval) for predicted 10-year cardiovascular disease risk greater than 20% (Panel B) among adults aged 30 to 74 years, by age group and food security status, National Health and Nutrition Examination Survey 2003–2008. Results are adjusted for sex, race/ethnicity, educational status, health insurance status, alcohol use, body mass index, and concentration of C-reactive protein. **Table:** 

**Panel A**			
**Age, y**	**Food Security Status**	**Mean**	**95% CI**
**30–59**	**Full**	6.5	6.2–6.9
	**Marginal**	6.2	5.1–7.3
	**Low**	5.7	4.8–6.5
	**Very low**	8.0	6.5–9.6
**60–74**	**Full**	19.1	18.2–20.1
	**Marginal**	19.9	16.1–23.7
	**Low**	18.3	15.0–21.5
	**Very low**	20.9	16.6–25.2
**Panel B**			
**Age, y**	**Food Security Status**	**Prevalence Ratio**	**95% CI**
**30–59**	**Full**	1 [Reference]	
	**Marginal**	0.92	0.48–1.77
	**Low**	0.94	0.53–1.64
	**Very low**	2.38	1.31–4.31
**60–74**	**Full**	1 [Reference]	
	**Marginal**	0.83	0.49–1.42
	**>Low**	0.89	0.56–1.41
	**Very low**	1.36	0.87–2.13

## Discussion

In this representative sample of US adults, food security status was significantly associated with predicted 10-year risk for cardiovascular disease among adults aged 30 to 59 years. Food security status was also significantly associated with several individual risk factors. As food security worsened, the prevalence of smoking increased substantially, and almost half of adults with very low food security were currently smoking. Furthermore, food security status was also significantly associated with concentrations of HbA1c and C-reactive protein among all adults as well as with BMI and obesity among women.

A few published reports noted associations between food security and smoking status (14–16). An analysis of data from the 2001 Panel Study of Income Dynamics found that among low-income families, the prevalence of smoking was 43.6% among food-insecure families and 31.9% among food-secure families (13). Data from NHANES 1999–2002 showed that children and adults who live in households with smokers were more likely to report food insecurity than their counterparts living in households without smokers after adjustment for age, sex, race/ethnicity, and household poverty income ratio (children: odds ratio [OR] for food insecurity, 2.0 [95% CI, 1.5–2.7]; OR for severe food insecurity, 3.1 [95% CI, 1.4–6.9]; adults: OR for food insecurity, 2.2 [95% CI, 1.6–2.2]; OR for severe food insecurity, 2.3 [95% CI, 2.4–3.7]) (14). Another analysis of NHANES 1999–2002 also described significant differences in the unadjusted food security status by smoking status (15). An analysis of data from Aboriginal participants aged 18 years or older of the Canadian Community Health Survey found a higher unadjusted prevalence of smoking among food-insecure participants than among food-secure ones (21). However, adjustment for sociodemographic factors substantially attenuated the association (OR, 1.20; 95% CI, 0.66–2.17).

The possibility that the prevalence of smoking is elevated among adults who are food insecure has several implications (13). To the extent that people who are food insecure avail themselves of government assistance programs, opportunities may exist to identify smokers and link them to resources to help them to quit smoking. Given the high cost of purchasing cigarettes, any reduction in smoking can free up funds that can be used for food purchases.

A review of studies in adult women and men suggested that food-insecure women were more likely to be obese than food-secure women in cross-sectional studies, but prospective studies generally did not confirm that food insecurity was associated with weight gain (5). Among men, no evidence supporting an association between food security and obesity was found. The results from the present study showing a significant association in women but not men are in agreement with previous findings from cross-sectional studies. To explain the association between excess body weight and food security status, it has been suggested that financially strapped people may purchase cheap energy-dense foods that are often high in calories (22).

The present analyses showed a significant association between food security status and concentrations of HbA1c but not prevalent diabetes once BMI was controlled for. The clinical relevance of a difference in concentrations of HbA1c of 0.15% between adults with full food security and adults with very low food security is uncertain. Several studies have examined the associations between food security status and prevalent diabetes or glycemic parameters (7,8,16). Among adults from rural Appalachian Ohio with a mean age of 44.7 years, no differences in mean concentrations of random blood glucose and HbA1c were reported (16). An analysis of data from adults aged 20 years or older who participated in NHANES 1999–2002 suggested that food insecurity was associated with self-reported diabetes mellitus (7). Participants with severe food insecurity had 2.2 (95% CI, 1.2–3.9) times the odds of having diabetes compared with participants who were food secure. Participants with mild food insecurity did not have a significantly elevated prevalence of diabetes (OR, 1.1; 95% CI, 0.7–1.6). A subsequent analysis of data from NHANES 1999–2004 again noted that food-insecure adults were more likely to have diabetes than food-secure adults (adjusted OR, 1.48; 95% CI, 0.94–2.32) (8). Because of constrained financial resources, food-insecure people are more likely to have low-quality diets that promote obesity and, ultimately, diabetes (23).

The results of the present study are subject to several limitations. The cross-sectional design of the study precludes establishing cause and effect for the significant associations. Second, the NHANES physical activity questions were changed for the 2007–2008 cycle, thus interrupting compatibility with the physical activity questions of preceding cycles. Consequently, physical activity was not included as a covariate in the study.

Food security remains a concern for many households. *Healthy People 2010* called for increasing the percentage of households who were food secure (24). The Community Food Security Initiative developed by the US Department of Agriculture is working to reduce the number of households that are food insecure (25). A host of community approaches can help to alleviate food insecurity in the United States (26,27). Recommendations for community strategies to improve availability of affordable healthy food and to support healthy food choices have been developed (28). Furthermore, some of the possible actions that can be taken by clinicians to assist their food-insecure patients have been outlined (29,30).

In summary, the present study found that the 10-year predicted risk for cardiovascular disease was increased among food-insecure participants aged 30 to 59 years, particularly those with very low food security. Furthermore, food-insecure adults were more likely to smoke, have a higher BMI or be obese (in women), and have higher concentrations of HbA1c and C-reactive protein than food-secure adults. Because little information is available about the cardiovascular health of food-insecure adults, additional research is needed in this area.

## Appendix. Questions Included in The Food Security Scale

Now I’m going to read you several statements that people have made about their food situation. Please tell me whether the statement was often, sometimes, or never true in the last 12 months.

1. “I worried whether our food would run out before we got money to buy more.”

2. “The food that we bought just didn't last, and we didn’t have money to get more.”

3. “We couldn’t afford to eat balanced meals.”

4. In the last 12 months, did you or other adults in your household ever cut the size of your meals or skip meals because there wasn’t enough money for food?

5. How often did this happen — almost every month, some months but not every month, or in only one or two months?

6. In the last 12 months, did you ever eat less than you felt you should because there wasn’t enough money to buy food?

7. In the last 12 months, were you ever hungry but didn’t eat because you couldn’t afford enough food?

8. Sometimes people lose weight because they don’t have enough to eat. In the last 12 months, did you lose weight because there wasn’t enough food?

9. In the last 12 months, did you or other adults in your household ever not eat for a whole day because there wasn’t enough money for food?

10. How often did this happen — almost every month, some months but not every month, or in only one or two months?
